# Circulars, Bulletins and Reports Issued from the Office of the Chief Surgeon of the American Expeditionary Forces in France

**Published:** 1918-11

**Authors:** 


### CIRCULARS, BULLETINS AND REPORTS

Issued from the Office of the Chief Surgeon of the American Expeditionary Forces in France.

*Under this heading will be published extracts from circulars and bulletins issued by the Chief Surgeon of the Medical Department of the American Expeditionary Forces in France. It is believed that these will be of general interest and value to medical officers.*

### EXTRACTS FROM C. S. O. CIRCULARS

### Pneumonia : its Prevention and Management.

*The Prevention of Pneumonia.* The present epidemic of respiratory infection in the A. E. F. is largely influenzal in character, with a rather high incidence of secondary pneumonia due usually to pneumococci or streptococci, and occasionally to influenza bacilli and possibly to meningococci. The mortality has been in the neighborhood of 30 o/o. As primary pneumonia is likely to increase with the advent of colder weather, medical officers are reminded that the prevalence of pneumonia, as well as of other respiratory infections in armies in the field, depends particularly upon :

1. Overcrowding.
2. Exposure to wet and cold.
3. Fatigue, whether induced by overwork, a long journey, loss of sleep, or nervous exhaustion from worry. Crowding forces the occupants in barracks or billets into close personal contact, and the greatest danger from it, in relation to the occurrence and spread of respiratory infections, is in the increased opportunity furnished from droplet infection of the healthy inmates from those who already harbor pathogenic micro-organisms in their noses or throats.

In epidemics of pneumonia or of influenza, the disease is undoubtedly usually spread from man to man through the secretions or discharges from the mouth, nose or other parts of the respiratory tract, and an individual who harbors virulent pneumococci, or streptococci, or influenza bacilli, is obviously very likely to infect his co-sleepers by coughing or sneezing, or even speaking loudly in close proximity to them.

In the present epidemic, the great majority of the cases of pneumonia are secondary to influenza, — the natural resistance of the individual having been first broken down by this disease, secondary infection of the respiratory tract with pneumococci or streptococci has occurred.

In Panama, where climatic conditions were not severe, pneumonia was prevalent, particularly on account of overcrowding, and the same was found to be true among the workers in the South African mines. Prevention consisted particularly in scattering the individuals and giving them separate dwellings in place of barracks.

*Overcrowding.* In relation to overcrowding, *Medical War Manual* No 1, for 1917, authorized by the Secretary of War under the supervision of the Surgeon General and Council of National Defense, states that whenever possible the floor space per enlisted man should be 80 square feet, affording 960 cubic feet, and should never be less than 10′×6′ or 60 square feet, which, with a ceiling 12′ high, would afford 720 cubic feet. This Manual further states that should an epidemic occur, and should the soldiers be overcrowded, it may be assured axiomatically that the epidemic cannot be checked by other sanitary measures alone, but must be combined with measures to relieve the overcrowding. Owing to the shortage of lumber and materials, it was thought necessary in the A. E. F. to reduce the space per man to one linear foot, or 20 square feet —one third of the minimum amount recommended. The order directs that bunks shall be 2′8″ wide by 6′6″ double tier, in sets of four, 2′8″ apart, giving one linear foot of Adrian barracks per man. It is hoped that conditions will soon be such that this allowance may be increased. In the meantime, an effort must be made to prevent droplet infection by other means between the men sleeping side by side in barracks. A board partition 2' high may be built between the two adjoining bunks. Until this is done, wires may be run two feet above the bunks, and the shelter tents suspended upon them between the adjoining bunks. Similar precautions should be taken in billets and tents. This is a more practical arrangement than placing the head to the feet of the adjacent sleeper. In cases where the overcrowding is excessive and the weather fine, the advisability of bivouacking the men in the open air under shelter tents, or other canvas, should be considered. If this is done, additional blankets obviously should be supplied. Relief from the dangers of overcrowding should be the first important consideration in connection with the checking of the present epidemic. Distance between beds is the important factor, not cubic space, in the prevention of the spreading of pneumonia infections. Crowding in recreation rooms, at cinematograph entertainments, etc., should at the present time be prevented as much as possible.

*Wet and Cold.* Wet and cold are also important predisposing factors in pneumonia epidemics. A lowered condition of vitality from cold favors particularly the development of such infectious diseases as pneumonia and influenza by lowering the resistance of the bronchial and pulmonary tissues to infection. Experiments suggest that infections with these diseases are favored by cold and chilling, through the stimulation of the mucous glands with resulting closure of the small bronchioles with plugs of mucus. It is well known that the functions of the leucocytes are disturbed by cold, and it seems likely that phagocytosis may play an important role in connection with the mechanism of immunity in pneumonia, and that immunity in this disease is particularly related to the functions of the leucocytes. The movements and phagocytic action of the leucocytes occur most favorably at about the temperature of the normal body. Exposure of the skin to cold and wet leads to chilling of the leucocytes during their repeated passage through the skin capillaries, which may diminish their functional activity and thus lower resistance to a point at which infection may occur. It should be borne in mind that cold wet feet produce a general reaction of the body, and not only a local one, and that this condition also predisposes to infection. Cold and wet have less unfavorable action when accompanied by energetic muscular exercises if a condition of fatigue is not reached. Additional effort should be made to provide for the prompt removal and drying of the wet clothing of the soldier, and additional blankets at night must be insisted upon.

*Fatigue.* It should be borne in mind that fatigue induced by overwork and also by lack of sleep and worry, in connection with wet and cold, has been one reason for the excessive mortality from pneumonia in armies in the field. It is well known that normal resistance to infection may be broken down by fatigue.

*Early Detection.* Greater attention should be paid by medical officers to the early discovery of cases of colds, cases of influenza and other respiratory infections, and to prompt isolation and treatment of such cases. Carriers undoubtedly play an important role in disseminating pneumococci, streptococci and influenza bacilli as well as meningococci.

*Warning Against Spitting.* Men should be specifically instructed at this time against expectorating in quarters, and the danger of sneezing and coughing and of speaking in close proximity to the face should be explained.

*The Management of Pneumonia.* 1. Pneumonia, especially as it occurs among troops, and as it is now present in the American Expeditionary Forces, must be regarded as a highly contagious disease, and it must be managed with the same precautions as are taken in the care of other contagious diseases.

2. The epidemics of influenza, now prevalent in many widely separated parts of France, have at least one point in common, i. e., the occurrence of pneumonia as an incidence of the disease, a complication, or a sequel. The pneumonia is usually of a patchy type, different slightly in its characteristics in different regions, but characterized by rapid progress, great respiratory distress, frequency of early collapse and high mortality. The causative organism may not always be the same; pneumococcus, streptococcus, the influenza bacilli and occasionally the meningococcus all seem to contribute their share.

3. Early isolation and hospitalization of pneumonia, as well as of influenza and similar respiratory infections, will do much to prevent the spread of the disease and lower the mortality. Cases should be hospitalized, when possible in medical formations, where they may remain until recovery, even though the initial trip by ambulance may be somewhat lengthened. Cases of pneumonia in the earliest stages withstand transportation fairly well, but later in the disease, after they are hospitalized, they are greatly injured by moving. Numerous cases of respiratory infection have been evacuated by train or by motor, arriving at their destination some hours later in profound collapse, and dying within a very short time. Moving a case of pneumonia to make room for a battle casualty may kill the pneumonia patient and not aid the wounded, and the practice should not be tolerated.

4. Isolation or segregation should be practiced in all cases of respiratory infection and such isolation should start in the field. Upon arrival at the hospital the cases of respiratory infection should be received in wards devoted to the observation of cases with respiratory infection, or, if it is possible to make an absolute diagnosis on admission to the hospital, the case may be sent directly to the ward designated to receive cases suffering from that particular type of infection. The observation ward for respiratory diseases should be cubicled, a sheet or other partition being placed between adjacent beds. It is desirable that an accurate diagnosis be made as soon as possible of cases in this ward, so that they may be transferred immediately to those wards designated to receive cases suffering from the different types of respiratory infections. All cases of uncomplicated influenza should be isolated in separate wards as rigidly as if they were cases of measles, and all beds should be cubicled. No cases of pneumonia should be sent to these wards, and should a patient with influenza develop pneumonia, he should be immediately removed to a pneumonia ward. Cases of pneumonia should be segregated in wards set aside for this purpose. These wards should be cubicled. The reason why such rigid isolation and employment of the cubicle system is imperative is due first to the fact that cases of influenza are highly susceptible to pneumonia, and may be infected with great readiness by a pneumonia patient in the near proximity; and second, that the lobular type of pneumonia may be caused by several varieties of organism, and should a patient with a pneumococcal pneumonia be placed next to one with a streptococcus pneumonia, either one or both patients might readily contract a double infection. The course of the disease in such double infections is much more serious, and the mortality much higher than in single infections. Cross infections will, therefore, be less common and the mortality reduced by cubicle isolation for all respiratory infections. The practice of receiving respiratory infections of unknown origin in wards with other medical or surgical cases is reprehensible, and is responsible for many fatal cases of pneumonia in individuals who might otherwise have been returned to duty within a short time. Cubicle isolation may most readily be carried out by screening with sheets. This can be done by posts and the use of wire, and can be adapted for tents as well as for wards. It is only necessary that the screen should reach midway between the foot and head of the bed, halfway between the bed and the floor and two and a half to three feet above the level of the patient. It is, however, highly important that the screen should extend several inches beyond the head of the bed.

5. Protection of medical officers, nurses, and personnel with gowns, and fresh, clean gauze masks is important, both to prevent spread of infection among them, and to prevent their transmitting infection to others. Attendants should be examined with the view to finding carriers. When found, carriers should be disinfected. All individuals who come in contact with cases of respiratory infection and fever should be masked, except in case of extreme urgency, and then unmasking should be offset by precautions to prevent the transmission of the disease to others. Patients should be masked while being moved.

6. Special attention must be paid to all cases of respiratory infection, with fever, with relation to the development of signs of pneumonia. It is often impossible at the outset to distinguish between cases of influenza, without consolidation, and actual pneumonia. All cases with fever, and with symptoms referable to the respiratory tract, must be viewed with suspicion and hospitalized, and the physical signs must be carefully watched.

7. Bacteriological examination in order to determine the infecting organism is important, not only from the standpoint of specific therapy, but also to facilitate the management of cases of different etiology. It must be remembered that pneumonia is really a group of diseases, with certain common signs and symptoms. The promiscuous mingling of cases of pneumonia, without determination of the infecting organism, is as harmful as the mingling of measles, scarlet fever and smallpox.

8. Specific therapy, when possible, is advisable. This will at present be limited to cases of pneumonia due to pneumococcus, Type I. The indiscriminate use of serum, without proper type determination, is ill-advised, not only on account of the fact that it subjects the patient to unnecessary inconvenience, discomfort, and possibly danger, but on account of the fact that serum is scarce, and must be saved for the cases in which it is actually indicated. The polyvalent serum may be used in Type I cases, as its titer for the Type I organism is as high as that of the monovalent Type I serum. The use of polyvalent serum in cases other than these due to pneumococcus, Type I, is not advised.

9. General treatment should be directed toward sustaining the patient and guarding against collapse. Under no circumstances should a patient with pneumonia, or suspected of having pneumonia, be allowed to walk, and after he is put to bed he should not be permitted to sit up for any reason whatsoever. He must be kept warm, but must be assured a continuous supply of fresh air. Fluids should be given freely from the start, and the patient should be induced to take them frequently and in considerable amounts. Sponge baths should be used to combat high temperatures.

10. Early cyanosis and collapse are characteristic of the present form of pneumonia. Treatment aimed to prevent and to combat circulatory failure should be instituted promptly on making the diagnosis of pneumonia. The early use of digitalis has been shown to reduce mortality, and is advised. It may be given in the form of a standard tincture, of which a total amount of 30 cc. (one fluid ounce) should usually be given. The following schedule may be followed.

If seen on the first or second day:

| | DAYS | | | | | | | | |
|---|---|---|---|---|---|---|---|---|---|
| DAY OF DIGITALIS THERAPY | 1 | 2 | 3 | 4 | 5 | 6 | 7 | 8 | 9 |
| Total amount of standard tincture . . . . | 5.0 | 5.0 | 0 | 5.0 | 5.0 | 0 | 0 | 5.0 | 5.0 |
| To be given in divided doses on the days indicated . . . . . . | MLXXV | MLXXV | | MLXXV | MLXXV | | | MLXXV | MLXXV |

If seen on the third day or later:

| | DAYS | | | | | | |
|---|---|---|---|---|---|---|---|
| DAY OF DIGITALIS THERAPY | 1 | 2 | 3 | 4 | 5 | 6 | 7 |
| Total amount of standard tincture . . to be given in divided doses, cc. . . | 10.0 MCL | 10.0 MCL | 0 | 5.0 MLXXV | 0 | 0 | 5.0 MLXXV |

The hospitals should supply themselves with a standard tincture of digitalis. Do not use pills which are dissoluble. Other stimulants, notably citrated caffeine and camphorated oil, may be used by hypodermic injection when collapse occurs or is imminent. The use of strychnine has not been shown to be of value.

11. Morphine is of great value to control severe coughing, to relieve the pain of pleuritis, and to secure rest for the patient. It should be used without hesitation. For the troublesome tympanites, that frequently occur, turpentine stupes, given while a small catheter is inserted in the rectum, are of value.

12. Most careful attention must be paid to the physical signs, particularly with relation to spread of the consolidation and to fluid in the chest. When the physical signs suggest fluid, exploratory puncture, with microscopic and bacteriological examination of the fluid obtained should be performed promptly. Exploratory aspiration is a simple procedure, with little danger or discomfort to the patient. Local anesthesia may be induced by freezing or by intra-cutaneous and sub-cutaneous injection of cocaine or novocaine. When clear or even slightly turbid fluid is obtained, even when the infecting organisms are demonstrated in the fluid, treatment by repeated aspiration with the Potain aspirator is followed by the best results; when purulent fluid is found, or in cases where fluid previously clear becomes purulent, operation is advised, with post-operative measures necessary to insur free drainage.

13. Convalescence must be managed with care, both as to the condition of the patient and as to his transmitting the disease to others. Development of pleural exudate late in the disease, or during convalescence, is not uncommon, and frequent physical examination must not be neglected. Relapse or spread may also occur after the temperature has been normal for several days, and the patient should not be permitted to sit up or move about until 7-10 days have elapsed. During this period isolation should be practiced as during the acute stage of the disease. The use of mildly antiseptic solutions in the mouth and nasal passages is of value in reducing the number of carriers. Patients should not be allowed to mingle with other patients, and should not be evacuated until all signs of infection of the respiratory tract have disappeared.

14. Recovery and return to duty will be slow. The final stages of recovery will best be provided for in convalescent camps. No patient who has had pneumonia should be evacuated to a convalescent camp until his temperature has been normal for at least two weeks, and in cases where the infection has been severe or prolonged this period will be materially increased. The patient should be free from cough, and other physical signs should be normal.

---

### EXTRACTS FROM C. S. O. BULLETINS

### The Experience of the American Expeditionary Forces with Respiratory Infections

Respiratory infections are understood to be those diseases which are passed by the moist discharges from the bronchi or trachea, the throat, and the nose and mouth, i. e., the spit, saliva and spray. Of the diseases included in the nomenclature of the Sick and Wounded reports of the A. E. F., the following eleven diseases are considered respiratory infections :

| A | B |
|---|---|
| 1. Mumps. | 1. Bronchitis (acute). |
| 2. Measles. | 2. Influenza. |
| 3. Scarlet fever (meningococcus). | 3. Pneumonia (lobar). |
| 4. Diphtheria. | 4. Tonsilitis (acute). |
| 5. Meningitis. | 5. Pneumonia (broncho). |
| | 6. Pharyngitis (acute). |

For pneumonia, as for the other respiratory infections, here as in civilian experience at home, crowding and close personal contact and exposure, while suffering from lowered resistance due to a variety of unfavorable conditions of occupation and environment, are apparently the dominant and in many instances the sole determining factors in causing the serious increase of respiratory infections among our troops in France.

Of the 1,613,500 days lost in the A. E. F. up to June 1st, 1918, from all causes — sickness, accident, injury in action and other traumatisms — 1,481,000 days or 91.78 o/o were lost because of sickness.

Of the total loss of days due to hospitalization from sickness, 631,226 days or 42.61 o/o were due to the 11 respiratory infections under consideration.

The striking effect of the epidemic of influenza, coming at a time of year when other respiratory infections are usually at their minimum, is shown in the sudden increase in rate in June.

*Causes.* Among the causes which are known to have contributed to the high incidence of the diseases in question are : Crowding in camps while awaiting, or in trains en route for, embarkation at U. S. Ports. Crowding, inadequate ventilation, insufficient medical inspection, and isolation facilities on transports. Crowding at ports of embarkation (in one instance the floor space per capita being only 8 sq. feet in barracks). Crowding in barracks throughout France, twenty sq. ft. and 200 cu. ft. per capita being the official allowance in regulation barracks with double-tier bunks. (British and French provision, except in front areas, being 40 sq. ft. and 400 sq. ft. per capita). Exhausting drilling beyond the point of reasonable fatigue. Working and sleeping sometimes in wet clothing and shoes. Eating of food served cold, and without shelter from cold and wet. Insufficient blankets for warm sleeping. Practice of awaiting complaint of sickness by men, instead of searching constantly for early cases of infections with coughs, colds and other early symptoms, and segregating them for observation, and for hospitalization, if necessary. Habitual neglect of open window ventilation of barracks at night in cold weather. Spitting promiscuously in and around barracks, mess-halls, kitchens, etc., and failure to protect the nose and mouth when coughing and sneezing among groups of men.

Some of the unfavorable conditions responsible for the high rate of respiratory infections are the inevitable result of the conditions of transportation on sea and land imposed by military operations, and the imperative requirement of speed in troop movements. Some are the result of limitations in labor and materials for the construction of shelter buildings for sleeping and eating. Some result from lack of imagination on the part of medical officers, whose experience has been chiefly in private practice where the prevention of disease in the individual from spreading to the community as a group of contacts has been subordinated to the treatment of the patient who comes with a complaint of symptoms. Some have been due to lack of discipline in matters of personal hygiene of the men in barracks, billets, and tents, owing to the indifference or disregard by line officers of the warnings and advice of medical officers. Whatever may be the limitations of space and shelter, conveyance or crowding, imposed by the prosecution of the war, there is much that can be done at once by a determined effort to remove every last accessory embarrassment or hindrance to the protection of the enlisted men of the A. E. F. from preventable respiratory infections.

*Barracks and Billets.* Wherever the number of men ordered to occupy barracks or billets exceeds one man per 40 sq. ft. of floor space, the following resources should be used : (*a*) Thinning out by providing tentage or makeshift outdoor shelter with increase of blanket allowance to those outside of buildings. (*b*) Alternate head to foot arrangement of men in bunks, with such supervision as will insure the observance of this order (the British have found it necessary to fix the bunks to the floor and lower the foot end of bunks several inches to insure observance of the head to foot order). (*c*) Where heads are unavoidably at same end of closely adjacent bunks, the arrangement of partition by wood or paper for 2 feet down from head of bunk to prevent spray infection.

*Preventive Measures.* Everywhere provision of bathing facilities with hot water; drying rooms for shoes and outer clothing; food served hot and under shelter from cold and wet; boiling water for rinsing cleaned mess kits. Policing of barracks regularly at night to insure free continuous flow of fresh air through open windows in all barracks and billets. Search for early symptoms and physical signs of catarrhal affections of eyes, nose, throat, and chest, and taking of temperature in suspicious cases, with segregation or hospitalization without waiting for well declared case of infection. Removal of common drinking cup from Lyster bags and other water supply points, and discontinuance of the habit of sucking water from the up-turned nipple of the Lyster bag. *Avoidance of drilling tired men especially when infection is prevalent and during periods of severe weather.*

*Influenza.* Report of a moderate sized epidemic of influenza, probably so-called " Spanish Grip ", in one battalion of the.... Infantry. Started August 22nd, and immediate steps were taken to isolate all men with fever or cough. Extra medical officers were sent out to take charge of the conditions, the number of billets were reduced, poorly ventilated billets were abandoned entirely, and it is believed the epidemic is under control.

With reference to prevention of pneumonia and other respiratory diseases, the condition of the billets all over the area is being investigated, and steps taken to reduce the number of men in each billet to a safe proposition in order that they may secure sufficient ventilation. The troops of this division are particularly prone to all forms of respiratory diseases.

*An Influenza Epidemic and the Environment and Care of the Men. From the... Division.* The conditions under which the men were billeted were extremely bad, the billets being in an ill-ventilated and dusty loft over a foul smelling stable. The men were working long hours under pressure, and returned at night always fatigued and wet from their work in the dug-outs. It is, however, interesting to note that the first case occurred on July 28th in the cook, and that the next two cases were also in the kitchen personnel. The third case occurred on Aug. 7th, and between the 9th and 13th the remaining 14 cases developed. About the 11th of the month the daughter of the woman who owned the billet, and who lived under the same roof, fell ill with the disease. The mother made the significant remark that she felt sure that her daughter had been infected from the troops because of the terrible *American habit of spitting*, that her court-yard has been kept constantly wet by the spitting of the men billeted in her stable. From the midst of surroundings such as the French peasants live in, this was an interesting commentary on one of the very few, but nevertheless very serious, breaches of hygiene practised by American troops. The billet had been evacuated as a result of the epidemic, which had not appeared in any other organization up to the time of my visit. The men were moved to another billet in a barrack building where they were housed under cleaner and less dusty conditions, but were crowded far too closely. In the course of our visit to the new barrack, the battalion surgeon and I found two cases in men who were not very ill, but who had been feeling miserable for about 24 hours. They said they were still undecided as to whether they would report " sick " or not, but they were sufficiently toxic to make it easy to pick them out among the other men. They were immediately removed and put to bed in the little advance infirmary.

In the management of epidemic diseases among troops at the front, this visit accentuates again the importance of *frequent inspection by the battalion surgeon.* In this particular instance, there is no doubt that the activity of the battalion surgeon in charge of this Engineering Company had much to do with the prompt recognition of the infection. It is, of course, not an easy matter to carry out regular inspections of troops living under front line conditions.

But it is not impossible. Battalion surgeons who are alert can do much to recognize early cases of infectious diseases. It means, however, eternal vigilance. More than at any other time, the battalion surgeon at the front becomes the first line of defense against epidemic disease.

*Recommendations* : That attention of Divisions Surgeons be invited again to the necessity of impressing upon battalion surgeons the need for constant vigilance in the matter of detecting early cases of infectious diseases among troops in the front line. That every effort be made before the approach of winter to start a campaign of education, and by every other means to diminish the spitting habit among our troops. This will require the co-operation of all officers and non-commissioned officers, both of the line and the Medical Department.

*Pneumonia Epidemic in Division.* The following extracts from a special report upon pneumonia and influenza in the.... Infantry call attention to the marked differences in susceptibility of new and old recruits to *the bearing of fatigue and personal habits upon the incidence of the disease,* as well as to practical remedies put into effect.

Report of epidemic of influenza and pneumonia at.... There are at this station 16 officers and 5210 men. The presence of an infectious febrile disease became apparent on August 18, 1918. It resembled influenza in onset and symptoms, but was of shorter duration. The prostration attending it was considerably greater than the height of the fever or the duration of the disease should cause. Coryza and signs of inflammatory conditions in the upper respiratory tract were marked. Skin eruptions were absent. There were no intestinal symptoms. The causative organism was apparently very active and virulent, for the disease spread rapidly. Pneumonia appeared about two days after the " influenza-like " infection. It has manifested itself both as a complication of the grippal condition and as frank lobar pneumonia, with typical onset, symptoms and signs. At the outset it was clear that we had *three factors operative in causing the epidemic.* (1) *The infection.* (2) *Overcrowding in billets, with poor ventilation.* (3) *Men unusually susceptible to infection on account of the following* : (a) *Unseasoned condition of soldiers.* (b) *Fatigue by long hours and an exhaustive training schedule.* (c) *Carelessness in regard to personal protection.*

Against the infective agent itself, we had no means of instituting a direct attack, so we were forced to try to overcome the factors which contribute to its spread. *Overcrowding was the rule in every*

*billet*. Ventilation, which was often poor at best, was usually less thorough than it should have been because windows and doors were frequently left closed. *Unnecessarily crowded arrangement of bunks was common*. The billets were too dusty. They had apparently never been sprinkled. This inspection was made after the men had gone to bed, as this is the only time when a satisfactory idea of the sleeping arrangements can be formed. Considerable re-arrangement was necessary in every billet.

The men did not have sufficient covering to insure warm sleeping. Bed sacks had been " turned in " according to orders. This necessitated the use of two of the blankets for a mattress leaving only one blanket and a shelter half for covering.

Swimming in the river after drill hours had been the custom. In spite of the fact that the water was flowing, it might, owing to the large number of men swimming in it, serve as a means for conveying infective expectoration and nasal discharge from one man to another. In addition to this, the water was very cold and it was probably too chilling to some of these men who were below par physically.

No special effort had been made to prevent men from congregating in crowded groups indoors. Nor had the value of remaining outside in the fresh air and sunshine, to prevent contracting " grippe ", been sufficiently impressed upon the men; not even light setting up exercises were being given in the morning.

*The hours of training prescribed by the present training schedule are too long for unseasoned troops*. This schedule allows sufficient time for sleep, but not enough is allowed for that recreative relaxation which is so necessary to overcome fatigue. The men come in from the day's training fatigued. They look and act " fagged ". Their condition is therefore such that any infection is easily contracted. It is the new men — those who have enlisted since May 1, 1916 — who are contracting the infection. All of the cases of pneumonia have occurred among these recently enlisted and, therefore, unseasoned.

In addition to not having the physical endurance of the average well-trained soldiers, *these recruits have been extremely careless about their personal habits*. Going to sleep on the floor without preparing the " bunk ", lying down on the ground after getting warmed up at drill, going swimming while still hot and tired, and *exercising no care about preventing others from coughing in their faces, etc.*, are some of the evidences of carelessness.

*Preventive Measures*. The following measures have been instituted to reduce the occurrence of respiratory diseases :

1. Re-arrangement of men's sleeping places.

2. Enforcement of orders requiring all doors and windows in billets to be kept open day and night.

3. Sprinkling of billets, including stairs and approaches thereto, every afternoon.

4. Recommendation that request be made that bed sacks be re-issued.

5. Discontinuance, for the present, of swimming in the common " swimming hole ".

6. Light setting up and deep breathing exercises after Reveille — just enought to warm the men up and stimulate the circulation.

7. *Instruction of officers and men in regard to causes and method of spread of respiratory diseases.*

8. The endeavor to " cull out " men who are in the earliest stage of the infection. This is done by having a medical officer visit the drill field about the middle of the day to hold an informal " sick call ". At this visit the officer attempts to separate incipient cases, which otherwise might remain with the other men and sleep in the billet with them until sick call the following morning. Another informal sick call is held at the infirmary after Retreat for the same purpose.

9. Advice to company commanders to make every effort to " cull out " from ranks men who are sick or are coughing excessively, sending them to the infirmary for medical examination.

10. Convalescent cases are to be held in the Battalion Infirmary for at least ten days after subsidence of fever — or longer if cough and expectoration persist. These convalescents are organized into a Sunshine Squad, and taken out doors on bright days for light setting up and deep breathing exercises.

11. Recommendation that the training schedule be modified to the extent of eliminating the fatiguing hours.

12. Arrangement of patients in the Battalion Infirmary into the following groups, with strict separation of the three : (*a*) Cases suspected of having pneumonia. (*b*) Cases of influenza. (*c*) Convalescents.

13. The billets shown by the epidemiologic map to be " infected spots " have been vacated temporarily. The men who occupied these will occupy shelter tents for a few nights.

— In the troops included in the above summary, the " old men " constitute almost half (45 o/o) of the total strength. Yet, in spite of this, pneumonia has thus far been confined to the " new men ". It is worthy of note that in Company " C ", which has only 32 o/o

" new men ", there has only been one case of pneumonia. Likewise, this company has by far the smallest number of men on sick report.

Upon the appearance of the first two cases of pneumonia, the Division Surgeon investigated, and found many cases in billets with fever, and evidence of influenza. Orders were given to the Local Surgeon to segregate all fever cases in one large billet, which was converted into a temporary hospital. The other billets were thinned out.

*Why wait? Pneumonia won't!* Thin out barracks and billets before instead of after an epidemic breaks out. Whenever a command is thoroughly shot with grippe or bronchitis, or there has been an outbreak of diphtheria and simple sore throats, it is found possible as well as desirable to get more floor space for the men in barracks, even to the extent of putting them under canvas, and in every instance with most favorable results. Shelter tent existence beats a sick bed. Men living in the open in the bitterest weather are uniformly healthier and especially freer from respiratory infections than are men housed in the same climate. See if you can recognize any of the quarters of the men of your command in this description of billets copied from an inspection of a divisional training area.

*Report of Conditions in a Training Area.* " The billet in which the influenza started was the ground story of an old house. 16 men were originally crowded into two poorly ventilated rooms. One of the rooms had only one window. 8 men slept in single bunks. In the other room 8 men were in two-tier double bunks. The ventilation of the whole place was poor. Opposite this, 52 men were quartered in a one-story and attic building with an adjoining stable. The men are now out in shelter tents. 27 men were billeted in a stable and loft, open only at one end by means of the door. Beds to accommodate 4 men in a row in two tiers were used for some of them. The rooms were low, dark and crowded. 9 men were billeted in the attic of an old French building with only one attic window; the place was dark and poorly ventilated. Beds were about one foot apart. 8 men were crowded into a dark attic room with one window partly below the floor. Ventilation was very poor. 42 man were billeted in a barn and loft with concrete floor. The bed sacks were nearly touching. Another billet in a barn was without a window, and the only ventilation was by means of the door. 26 men were billeted in another barn, one of the rooms of which was unlighted and unventilated except by a door partly below the surface of the ground. "

*Bacteriology of "Spanish Influenza".* Der Bakteriologische Charakter der "Spanische Krankheit". *Deutsche Med. Wochenschr.*, Berl. u. Leipz. 1918. 4, 775, 808 and 863. Editorial Notes, *Munchen. Med. Wochenschr.*, 1918. 68,804.

The pandemic of influenza has not spared any single part of Germany. The clinical course does not seem to differ from that run by the disease in this country. Measles and fatal pneumonias are particularly noted. The clinical picture is declared to be identical with that of the last pandemic of 1889. A very striking observation was brought forward, and generally confirmed, at a special meeting of the Munich Medical Union on July 9th, namely, that it is mainly persons under thirty years of age who fall victims to the disease. This was explained by a survival immunity in the elder generation. The meeting considered all the aspects of the epidemic on the basis of the hospital and university material of Munich. Pfeiffer's bacillus has been found only exceptionally. Streptococci and occasionally pneumococci were recovered from the sputum, organs, and blood of the patients. Similar findings were recorded in 1889, and thus the present results were in "keeping with precedent". Pfeiffer's bacillus had not been found until 1892, although it would have been impossible to overlook it in 1889. Thus, it may be that it will yet turn up in due course. Pfeiffer has not established the relationship of the present epidemic with the infection of the pandemic of 1889-92. He finds the bacillus influenza in current cases, but not uniformly. Gruber of Munich says "Influenza bacilli not found yet — investigations proceeding". Friedemann of Berlin finds symptomatology and complications correspond exactly with those of 1889-90. He has not found the Pfeiffer's bacillus — streptococci and pneumococci being the most common agents of the complicating pneumonias. Uhlenhuth, so far, has reported from Strasbourg the same contradictory results as those of Pfeiffer.

The University clinic of Budapest telegraphed on August 1st, that the bacteriological investigation of some 200 cases demonstrated the Pfeiffer's bacillus as the cause of the outbreak.

Kolle reported, under the date of July 18th, from Frankfort, his failure to detect Pfeiffer's bacilli in any of the few cases which he had thoroughly examined. In practically all cases, however, there were found large numbers of a Grampositive coccus — often in a pure culture or in symbiosis with pneumococci. The diplococcus tended to develop involution forms, and to grow in very long chains in the condensation water. He regards them as agents of a secondary infection in the "Spanish disease" which, to his mind, may not be identical with the pandemic influenza of 1889 to 1893.

*Present Situation in the A. E. F.* During the past week there has been further extensive incidence of pneumonia, in almost all instances accompanying the prevailing epidemic of influenza. Large convoys brought to the Base Forts several thousand cases of severe influenza, and many hundreds of cases of pneumonia. The death rate (case mortality o/o) among the pneumonias has risen to 32 o/o for the A. E. F., and in some groups of cases it has been 805.

Where unseasoned men have been sent to the A. E. F., the percentage attacked by influenza on shipboard, and the percentage of complicating pneumonias developing among these contingents after arrival has been unusually high, 100 o/o sick being reported from the 539th Labor Battalion (negro).

In addition to the three cardinal causes of high incidence of respiratory affections, which still prevail widely, (i. e., 1. overcrowding; 2. exposure to wet and cold; 3. fatigue from over-work, long journeys, loss of sleep or worry) there has been much pneumonia developing among men with influenza evacuated to Base Hospitals, who should at all costs and by every means possible be kept where they are taken sick. Absolute rest, even though in the absence of skilled nursing, or under very primitive conditions of shelter, is indispensable to escape the complication of pneumonia.

Meningitis has made another sudden rise in incidence, as had been expected. Forty-eight of the seventy-one cases reported in the week were in the Base sections, and thirty-three of the cases were found among troops on arrival of a convoy at one port.

Dysentery, diphtheria, measles, paratyphoid, scarlet fever and typhoid fever show a very satisfactory reduction during the week.

### Judgment and Ingenuity Demanded in Transferring Cases between Hospitals.

(*a*) Do not transfer pneumonias or respiratory tract infections. Absolute rest is as vital to them while they are meeting and overcoming the infection as operation is for a penetrating wound of the abdomen. Infectious diseases while still in the communicable stage should not be transferred.

(*b*) A good many cases of bone and joint injury are being evacuated from one hospital to another, either inadequately splinted or entirely without splints. The excuse commonly given is that standard splints are not available.

Such an excuse is, of course, entirely insufficient, since even hough the standard splints are not available, the ingenuity of the surgeon should be used to devise some form of appliance that would be reasonably satisfactory for transport use until the regular splints can be used.

### Pneumonia.

Septic pneumonia, due commonly to the streptococcus haemolyticus, has appeared in virulent form in a dozen or more different and widely separated points in France. There has been a high mortality (20-40 o/o), deaths occurring in many instances within 24 and 48 hours of onset of symptoms. The conflict between infection and resistance, once the symptoms are well declared, is apparently but little modified by any treatment provided.

Exhausted, driven, anxious men are easy prey to infection. The condition of the man when exposed to infection is of far greater importance than the care he gets after he is sick in bed. A man is entitled to at least the devoted care given a horse. The man's resistance is largely at the mercy of his officers, and to them we must look for the greatest measure of prevention.

Here is what energetic and resourceful medical men are doing about the pneumonia situation in the A. E. F., through improvements in sanitation of environment and control of personal habits.

*Pneumonia a Contagious Disease* (*From Headquarters, 52nd Division, Office of Div. Surgeon*). For the purpose of prevention and treatment, pneumonia will hereafter be regarded as a contagious disease by the medical personnel of this Division.

If a case occurs during an inactive period, or while in billets, the following will govern :

1. Send patient to hospital designated.

2. Report the diagnosis to this office by telephone or courier, giving name, number, company, organization, number of contacts and where they may be found.

3. The contacts will be segregated. The term Contacts will be understood to mean all those who have been in close and more or less permanent relationship with the sick man, those who sleep in nearby and adjoining beds, and those who work with him in kitchens, offices, etc.

4. The Surgeon will immediately make an inspection of quarters, reporting number of occupants, cubic feet of air, light, etc. He will arrange for a thorough cleaning and disinfection of the billet, and institute efficient preventive measures such as antiseptic treatment of the nose and throat of all contacts.

5. Upon arrival in hospital, the case will be isolated, so far as practicable, by screening or otherwise. Attendants will wear gauze masks over nose and mouth and will be given the same treatment as other contacts.

6. One or more wards, as necessary, will be designated as Pneumonia Wards. No other cases will be admitted.

7. No visitors will be allowed, except when absolutely necessary.

8. Utensils, linen, etc., will be handled as with other contagious diseases.

9. Pneumonia, as with other respiratory diseases, is spread by careless expectoration and coughing (droplet infection). Effective regulations must be enforced for prevention of infection by this means. Educate the men as to the dangers of careless expectoration.

10. Battalion Surgeons will make frequent inspections of billets, and inspections and examinations of men, in order personally to know at all times the conditions under which the men are living and their physical condition. The Battalion Surgeon who does not know the physical condition of the men at all times is *not* giving efficient service. The daily sick call does not give sufficient opportunity to know the true condition.

Pneumonia is a disease, the result of human contact. It is spread by over-crowding in billets, by exposure to cold and wet, by exhaustion, etc. See that the men have a reasonable air space in billets, and that the billets are ventilated. Sleeping with heads placed alternately prevents men from coughing in each other's faces.

The effective strength of this Division depends to a large extent upon the earnest efforts of all medical officers to improve sanitary conditions. Where such efforts do not bring results, submit report to this office stating action taken, dates, and names of responsible officers, in order that the matter may, if necessary, be brought to the attention of the Commanding General.

*Evacuation or Transfer of Pneumonia Patients.* Medical officers are warned against exposing pneumonia, or patients with severe pulmonary disease with fever, to the fatigue of transportation, unless under urgent necessity for space for battle casualites. Complete rest in pulmonary disease is absolutely indispensable.

### Error of Diagnosis or Poor Judgment in Evacuaion? Reported from Hospital Center.

Private..., ...Infantry, received with a well developed attack of lobar pneumonia but with a diagnosis of influenza from Base Hospital. Private P. B., Medical Department, 18th Infantry, arrived with well marked pneumonia from which he shortly died, transferred from Base Hospital. Private..., ...Infantry, well developed broncho pneumonia on admission from Base Hospital

### Influenza, Pneumonia, Meningitis.

During the past two months a second wave of severe influenza infection has swept over France and has spread to all the countries of Europe in about equal force. In the United States the onset of the epidemic was, as is usually the case with pandemics of influenza, about three weeks later than in London and Paris. The first and rather benign phase of the infection, it will be remembered, began in the middle of April and had largely disappeared in the A. E. F. by the end of July. The second phase, which has not yet reached its maximum incidence, has been characterized by a much higher percentage of initially severe cases and particularly of pulmonary complications. Coming at the time of rainy and changeable weather, this new invasion of infectious colds and coughs has been accompanied by a constantly increasing number of pneumonias. Now replacement draft detachments arriving with each convoy have added the heaviest percentage of infected men per strength and have shown the highest percentage of complicating pneumonias. It has been a usual observation that when infections of the upper respiratory tract prevail, the incidence of meningitis in the community increases soon after, and this rule prevails at present. An increasing severity of the pneumonia is commonly found when the disease is permitted to pass rapidly through successive hosts.

It is known that not less than 200,000 cases of influenza were reported during the past week among the troops in the camps in the United States. It has been officially reported that 80 o/o-90 o/o of the mobilized army of Switzerland was affected soon after the introduction of the infection by prisoners exchanged through Switzerland from Germany. From official information from Portugal it appears that the influenza infection is no less severe there than in the A. E. F. and the pneumonia is of as fatal a type.

The influenza and pneumonia now prevalent among French civil and military population is at least as severe as in the troops of the A. E. F. The areas of heaviest infection of influenza, pneumonia and meningitis in the A. E. F. are the Base Ports, the Depot Divisions and such training areas in both S. O. S. and Advance Zones as have received replacements or new organizations still including men exposed to the massive infection which has prevailed on the transports and on troop trains.

Substantial relief is to be expected soon from the frequently recurring infections introduced through Base Ports by incoming troops by the following improvements :

(*a*) Careful exclusion of men with colds, coughs and fever from transports at ports of embarkation;

(*b*) Equipment of all troops prior to embarking with three blankets, an overcoat, and winter weight woolen underclothing;

(*c*) Reduction of troops carried on transports to 80 o/o of berth capacity;

(*d*) Increase of hospitalization capacity on transports to 4 o/o of troops;

(*e*) Shelter ready and standing for troops on arrival at Base Ports, with provision for permanent kitchen and mess service for arriving troops;

(*f*) Period of not less than 4 days with no heavy duty on debarking;

(*g*) Medical supervision of troops on troop trains;

(*h*) Gradual hardening and acclimatization process at Depot Divisions, with isolation so far as practicable of new arrivals from earlier arrivals or permanent troops until infection has been eliminated;

(*i*) Increase in the floor space per capita to be provided wherever practicable up to 40 square feet per man;

(*j*) Separation of adjacent bunks by permanent board or shelter-half partitions.

It will take continued concerted effort by all medical officers in the application of all measures of local sanitation and supervision of the personal hygiene of the men to avoid further extension of influenza with its complicating pneumonias and often coincident meningitis. Men's bodies must be kept warm. Their clothing must be dried at least once a day.

Sufficient blankets and drying rooms accessible to every one especially in the regions immediately back of the fighting front are indispensable for prevention of pneumonia. In the S. O. S. and in areas occupied by troops in training or reserve the problem is largely one of personal contact and crowding; among the troops at the front it is a question of fatigue and exposure; determination to remedy both will go far to save lives.

A report from the First Army Corps indicates that pneumonia is not epidemic, though prevalent there; that influenza bacillus and pneumococcus are the usual infecting bacteria; that incomplete clothing equipment and sleeping in cold and wet are the chief contributing causes.

*Resources when Barrack Space is Cramped.* (Surgeon, Base Section n° 5).

Shift hinges on windows at barracks, where bunks do not prevent, so that sashes open inward and circulation of air from entire window area is obtained. At least four windows can be so fixed and kept open in all weathers, day and night. Open alternate windows full length 9 a. m. to 5 p. m. daily. Keep men from using bunks as day-time lounges.

*Barrack Rules of 312th Field Artillery.* (5) That the N. C. O. in charge of each room in the barracks be made responsible for the ventilation and cleanliness of his part of the building, and that the following rules apply :

(*a*) That the top transom be kept open at *all* times, without reference to the weather;

(*b*) That all windows be kept open at night, except when there is beating rain or snow;

(*c*) That stoves be banked at night, and that at all times a can of water be kept thereon;

(*d*) That the floor be dampened before sweeping;

(*e*) That all articles of clothing and equipment not used daily be placed in barrack-bags, so that the germ-laden dust may not collect thereon;

(*f*) That the woodwork be wiped twice weekly with a dampened cloth;

(*g*) That bedding be aired and sunned twice weekly, weather permitting;

(*h*) That clothing be brushed, shoes cleaned, and blankets shaken *out of doors.*

*The Susceptibility of Gas Cases to Respiratory Infections.* (N° 1, Presby. U. S. A, General Hospital, B. E. F. France).

" At the Red Cross Meeting on October 5th, 1918, the subject of the susceptibility of individuals suffering from measles and influenza to pneumonias of the streptococus and pneumococcus origin was discussed, together with the frequency with which normal individuals in the A. E. F. harbor streptococcus. Poison gas of the lung irritant type is another most important cause of susceptibility to respiratory diseases. This was not brought up at this time and should be accentuated.

During the last year and a half, it has been noted here in the B. E. F. that respiratory infections in gas cases are much more frequent during the winter than during the summer months and that, by segregating gassed patients from all contact with respiratory diseases, the incidence of secondary infections could be markedly diminished. Whole convoys of gas cases which were not properly protected have soon become secondarily infected.

The mortality in the B. E. F. is very low from these respiratory infections owing probably to the relative absence of any virulent respiratory disease, but even the mild cases of bronchitis were delayed from two to six weeks in their return to Con. Camp.

It seems almost certain that, with the prevalence of virulent streptococcus, pneumococcus and influenza in the A. E. F., the incidence of fatal secondary infections will be much higher than in the B. E. F. With this in mind, it would seem highly advisable to mask all gas cases with gauze when first seen, to prevent secondary infections and to spray their upper respiratory tract in the hope of diminishing what infections there might be present. A solution of menthol, phenol, camphor, and eucalyptol in albolene has been found to be very soothing to the burned mucous membranes and very efficacious in diminishing these secondary infections ".

*Patients with pneumonia* must not be moved. Case in point : Lt. H. G., 7th Inf., admitted to Base Hospital 23 from Evacuation Hospital 7, via Hospital Train 53, October 11th, and died of pneumonia the same day at 11.30 p. m. Diagnosis tag and Field Medical Card read : " Oct. 9, admitted to Evacuation Hospital 7. Influenza of 8 days duration. On October 11 at Base Hospital 23 the diagnosis on admission was broncho-pneumonia ". Influenza still seriously ill after 8 days is almost always a case of pneumonia.

Meningitis also is not suitable for transfer or evacuation during acute febrile stage of the disease. Base Hospital 23 received Pvt. A. A., 314th Inf. and on Oct. evacuated him by French Sanitary Train. At 6 a. m. on October 17th patient was admitted from the train to Camp Hospital 28 with cerebro-spinal meningitis.

### Decalogue For Disease Defense

" *Ten Commandments for Keeping Well.* (Hospital Center Saveney).

*Sweeping.* The law already in vogue against dry sweeping must be enforced : *(a)* In the wards by the nurses in charge. (*b*) Outside of the wards by the Sanitary Officer.

*Spitting.* Officers, nurses and other authorities of the Center are to report to the Adjutant any person breaking the ordinance affecting this subject.

*Sneezing and Coughing.* All persons sneezing or coughing must hold a handkerchief or piece of gauze before the mouth. Gauze will be provided by nurses in charge of wards for this purpose.

*Gatherings.* Persons who cough are to be excluded from any indoor gathering and from the Y. M. C. A. huts. Officers and

Y. M. C. A. authorities present shall be held responsible for carrying this out.

*Sputum Cups.* Ward masters shall be hold responsible for the cleanliness of sputum cups. Special emphasis is to be laid on the cleanliness of the outside of the cups.

*Mouth Hygiene.* It shall to the duty of the nurse in charge of the ward to know that every patient in her ward has brushed his teeth with tooth powder or paste at least once a day.

*Drinking Cups.* The use of a common drinking cup is forbidden.

*Clothing of patients.* Patients leaving the wards must wear underclothing, O. D. shirt, breeches, shoes and socks. Blouses or overcoats or both are to be worn when, in the opinion of the ward surgeon, the same are necessary. Ward Surgeons will say what clothing the men are to wear each day. Dry clothing must be insisted upon when patients return to the ward with wet shoes, stockings or outer clothing.

*Common Colds.* Cases of common colds are to be examined carefully and by ward surgeons to exclude more serious respiratory conditions.

*Instruction of Patients.* Patients are to be instructed by ward surgeons and nurses that to keep dry, to keep the feet warm and to obey the above commandments mean the health of all concerned."

*Recommendations Made by Sanitary Inspector of the Advance Section and Issued to Post Commandant, A. P. O. 704, by Commanding General, Advance Section S. O. S.* :

*Barracks and Billets* : (*a*) Barracks and billets will be thoroughly ventilated weather conditions permit, by keeping the windows open;

(*b*) During bad weather only those windows to be closed that are needed to keep out the rain;

(*c*) At least 40 sq. ft. will be allowed for each man both in barracks and billets, and if it be necessary, to insure this amount of space by using corridors for beds and by moving part of the command into tents;

(*d*) The beds of the men will be arranged alternately head and foot;

(*e*) All bedding will be sunned and aired for two hours daily whenever the weather permits;

(*f*) Men will not be allowed to congregate any more than necessary in barrack, and they will not be allowed to sit on the beds of the other men;

(*g*) Men will be prohibited from spitting on the floor;

(*h*) Spittoons will be provided containing sawdust, and the sawdust be burned daily;

(*i*) Men will use their own cups for drinking.

*Limited Quarantine* : (*a*) Men will not be allowed to visit hospital buildings or tents or any other buildings than their own quarters in the post or outside the post except in the performance of their official duty;

(*b*) All cases will be promptly isolated and no unauthorized soldiers will be allowed near the patients;

(*c*) Convalescent patients will be strictly confined to the wards, and they will not be returned to their commands until one week after all symptoms have subsided, and under no conditions in less time than ten days after admission to the hospital;

(*d*) Officers and soldiers will be restricted to the immediate vicinity of their billets, and prohibited from visiting other towns or camps except on official business;

(*e*) Officers and soldiers entering the camp from any other billeting towns on official business will be required to stay in open air;

(*f*) Officers and soldiers in the camp will be kept away from the billeting areas;

(*g*) All officers and soldiers will be kept away from civilians except in the performance of their official duties.

*Instruction of the Men* : (*a*) The men will be instructed as to the method of transmission, the highly infectious character of the disease, and the liability of infection by proximity to others;

(*b*) The men will be instructed that a man may transmit the disease, although he does not feel sick enough to go to the hospital, and ultimately be responsible for many cases of infection;

(*c*) The men will be instructed to report immediately when they begin to feel sick, and to avoid coughing in the direction of others.

*Inspection by Medical Officers* : Organization medical officers will inspect every member of their respective commands late in the afternoon or evening, and isolate all suspicious cases.

*Protect the Little Children of France in billet areas by weeding out the tuberculosis soldier promptly.*

" The communes of France of 5000 or less inhabitants have less tuberculosis than any comparable villages or small towns in the world.

The children (few enough already) in France must not be exposed to our tuberculosis soldiers.

A consumptive soldier, through close contact in billets, can infect through massive doses the healthy soldiers ".

*Then also* : " From a study of the tuberculosis cases recently reported it is evident that the tuberculosis soldiers are not being picked out soon enough to give them a chance for recovery.

It is noticed that the officers are picked up quicker than the men.

In some of the armies engaged in this war it has been estimated that possibly not 10 o/o of the tuberculosis soldiers have a chance for recovery.

There is an opportunity to demonstrate to the world that if the tuberculous wounded are picked up more promptly they will be the more curable.

Eradicating tuberculosis is like weeding a garden; there are always more cases to be eliminated.

Quiet times in Base Hospitals can be well utilized by examining personel and nurses as well as surgical and medical cases for pulmonary tuberculosis.

Quiet times in Divisions may be utilized by regimental surgeons in ferreting out the tuberculous cases."

### Pneumonias and Bronchitis Mistaken for Pulmonary Tuberculosis.

Many slowly convalescing pneumonias and broncho-pneumonias and sub-acute bronchitides are being mistaken for cases of Pulmonary Tuberculosis in the A. E. F. Grippe and the pneumonias frequently leave a residual fraction which may last for years and never be entirely removed.

*Symptoms.* Chiefly cough and expectoration; the sputum is negative to tubercle on repeated examinations. The patient appears well but may have occasional bouts with fever lasting several days. In later life these men, if not properly handled now, will become sub-standard with a loss of about 20 o/o body weight.

*Physical Signs and Examination.* Usually moist rales of typical indeterminate types, (showers coarser than subcrepitants) are present either at bases or at times between clavicle and nipple. Oftentimes there are no physical signs on ordinary examination even after cough. Examination of the soldier in the inverse position will reveal those rales in the lower lobes posteriorly or the lower parts of either upper lobe anteriorly.

*Inverse Position.* Have soldier kneel on chair; place his hands on floor close to the chair; the head hangs down like a pendulum. With back straight the man is then upside down. Practice for increasing lengths of time will enable the soldier to gradually remain in this posture five to fifteen minutes until the lung toilet of complete emptying is obtained. *Examination* should be made in this position.

*Treatment.* The inverse position should be assumed every three or four hours at first; later, when cough and expectoration avoided, every six hours. The foot of the bed can be sufficiently raised in some cases to cause continuous drainage.

*If drainage is applied early such types of lung infection by this treatment may avoid life-long Bronchiectases.*

Base Hospitals and Convalescent Camps will save bed space by early adopting these principles. Many soldiers can thus be salvaged from Classes C. and D.

### The Campaign Against Winter.

A Contribution from the Surgeon of the Fifth Division, A. E. F.

I. The troops of the American Expeditionary Forces are entering upon a winter campaign. Our enemies are the Germans and the cold. Of these two enemies, the cold is the more formidable. If we do not fight him in the right way, General Winter will injure and kill more of our men than General Hindenburg. We can fight the cold as effectively as we can fight the Germans, if we only use the right methods. General Winter need claim none of our victims. We must, however, perfectly understand what we have to do and the methods which we must use to keep ourselves strong and well.

For fighting the cold we must have plenty of good, well-cooked food, warm dry clothes, shelter and heat. If those necessities are provided, the winter campaign will be an invigorating experience. Without them a winter campaign will bring sickness, death, defeat and disaster.

Men must be supplied with the articles which they need for fighting the cold. They must be trained in the use of those articles, just as they are trained in the use of offensive weapons. It is as important that the soldier keep his feet dry and his body warm as it is that he has his rifle in working order.

Soldiers who have poor food, insufficient or wet clothes and no shelter during the cold weather, have no more chance to live than shorn sheep in a Montana blizzard.

Our troops must be supplied with the articles which they require and then must be taught that their lives depend upon the care and proper use of their equipment. They must have detailed training in care of clothing and in the methods for providing themselves with temporary shelters until materials for better protection can be brought to them.

Food is the fuel which warms our bodies. The stomach is the stove in which it burns. The arteries and veins are the pipes which carry the warm blood to every part of the body and so keep us from suffering from cold.

Our clothing furnishes part of the shelter which keeps the heat from escaping from our bodies. When we do not live in warm houses, we must wear more warm clothes. We must learn how to make small shelters and huts such as are made by trappers and travellers in cold countries. We must learn to make the best of small fires and of the simplest means for keeping ourselves and our clothing dry and warm.

II. Cold takes heat and strength from the body. If the cold is sufficiently intense and there is but little protection against it, the soldier will be frozen to death in a short time. This may happen during severe weather.

The slow effects of less severe cold, though less obvious, are more dangerous. By long exposure the soldier becomes weakened so that he falls a victim to any sort of infection which may be present. Because of weakness from exposure to cold we may have epidemics of Influenza, Diarrhoea, Meningitis or Pneumonia. Persons who have latent tuberculosis may develop quick consump tion.

When men cannot or do not fight the cold in the right way, they become uncomfortable and miserable. They lose their courage and fighting spirit. They sicken and die. The fighting unit first loses its efficiency and then loses its men.

Disasters from cold are not necessary. They come from neglect of duty on the part of Supply Officers, unit Commanders or individual soldiers. If the plain duty of any of these individuals is neglected, the soldiers suffer, and the war is prolonged.

III. *Crowding.* Crowding is dangerous. During cold weather all animals tend to huddle together for the sake of warmth. Large numbers of men crowd into small shelters. A direct shell hit on such a shelter may kill them all.

There is, however, the greater danger which comes from their infecting each other with epidemic diseases. When men crowd into small rooms, huddle about the stove and expectorate at it, they give each other influenza and pneumonia. The close, damp, hot air becomes filled with germs of disease.

Have many small shelters and many small fires. Keep the air pure and the hut and stove clean. Throw trash into the fire and not at it.

IV. *Food.* Since our food is the fuel upon which we must rely to give us heat and strenght, it is absolutely necessary that the food supply must be ample, that it must be good, that it must be furnished regularly to every man, that it must be well cooked and well chewed and digested.

It is the duty of every supply officer to see that plenty of food is furnished to each unit. It is the duty of each unit commander to see that each man in his command gets well cooked food. It is the duty of each individual soldier to chew his food thoroughly and eat it slowly, so that his stomach will be able to digest it. Unit commanders must pay particular attention to their kitchens, to see that the food is clean and well cooked and that none of it is wasted. Particular attention must be given to the use of meats. Do not allow the fat to be thrown away. Large beef bones must be broken and used in soups and stews, as the food value contained in marrow and joint tissues is of great value in keeping up the heat of the body.

Soldiers who have indigestion must be sent promptly to the surgeon, as simple remedies will often enable them to digest their food and keep themselves strong.

In order to maintain physical vitality of men who are exposed to the elements, it may be necessary to feed them frequently. Certain British trench orders have required that a hot meal be served to men in the trenches between twelve, midnight, and four in the morning.

V. *Clothing.* The clothing supply must be ample. Each soldier needs one warm cap, one overcoat, one uniform-blouse and breeches, one woolen shirt O. D., one suit of heavy underclothing; and, in cold weather, two suits, both to be worn at the same time, one pair of heavy gloves or mittens, two blankets, four pairs of socks, two pairs of large thick shoes, and one pair of leggins. To this may be added one Red Cross sweater, one shelter tent half, and one poncho or slicker.

Wet clothing is not warm. When clothing becomes wet and damp, heat and strength leave the body. The soldier becomes chilled. He may be frozen to death or he may become so weakened that he has not enough vitality to keep him free from sickness. We must keep ourselves strong by keeping our clothes dry,

There are many way of drying clothes:

(*a*) The best and simplest is by use of the sun and wind. Have all damp and wet clothing sunned and aired on every clear day. Many men get sick because they have their blankets folded up on their bunks and do not dry them when they have a chance.

(*b*) Clothing may be dried by hanging on wires or cords near kitchen or barrack stoves. It is often an easy matter to dry clothes in this simple way.

(*c*) Simple and effective clothing dryers may be made as follows: Make four screens 6′ × 5′. These screens are covered with burlap. Old gunny sacks are good for this purpose. The frames of the screens may be made of lumber if it is available. If cut lumber is not at hand, the frames may be made of poles lashed together at the corners. Those screens are set together to form a square with a Sibley stove in the center. Clothing hung inside of such a frame is quickly dried. In one such frame, a coat which had been soaked in water was dried in twenty-five minutes.

(*d*) A simple way of drying socks is to put your hand inside them and hold them near the fire. Many people have kept their feet from freezing by use of this simple method.

(*e*) Troops are to be supplied with mobile laundries at the rate of two for each division. Each of these laundries has a dryer which may be used for drying some of the clothing.

It is often very difficult to keep the clothing properly dry. Constant attention must be given to this matter and every means available must be used to the fullest extent.

VI. *Feet.* The most important articles of clothing are those which cover the feet. When the feet are wet and cold the men become tired and miserable. They lose strength and vigor. If this condition continues, they will become sick, or will get "trench foot". Men who wear big water-proof shoes, and who keep their feet dry, never get "trench foot". Men who are strong and vigorous, and who have good circulation of blood through their feet, do not get "trench foot".

Shoes must be very large. They must be big enough to hold the feet and three pairs of heavy woolen socks without pressure upon the feet, which will prevent circulation of the blood. Shoes must be thoroughly greased with "dubbin". One application is not enough.

The leather must be thoroughly soaked with "dubbin" and repeated applications must be made. To be useful, "dubbin" must be used. It is the duty of unit commanders to see that it is used by every man in the company, and that shoes are kept waterproof, soft and pliable. Constant attention must be given to drying of socks.

When the feet get cold, the quickest way to warm them is by using them. A short run will speed up the circulation of blood through the body, and render warmth to the feet. If the conditions are such that the troops cannot run or march briskly, they may use other active exercise to bring up the circulation. Brisk exercise is the simplest and surest way of restoring warmth to the body.

Where men are obliged to stand for hours in mud and water, it is often impossible to keep the feet warm. It has been found that a thin coating of oil or grease applied to the legs and feet helps to keep the heat in them and to prevent " trench foot ". Rubbing the foot is useful. In cold weather shoes and stockings may be removed once or twice daily. The feet are then rubbed briskly and a thin coating of oil, " dubbin ", or other grease may be rubbed into the skin, after which dry socks and shoes are put on.

VII. *Shelter.* During winter campaigns armies may be required to operate in a country which has been devastated by the enemy. There will be no barracks or buildings left standing. It may be impossible to use conspicuous tentage. There may be objection to assembling men in large groups where a number might be killed or injured by a single shell.

Men must be taught how to make small, comfortable shelters for themselves. These huts and shelters may be improvised from materials which are to be obtained where the division is operating. They cannot be conspicuous nor large. When troops are moving, no elaborate shelters can be made.

The first essential of a shelter is that it protect the soldier's bed from water, snow and wind. The second essential is that it be provided with a place for a fire. Other materials are luxuries, and not essentials. The kind of shelter which is made must depend upon the materials which can be had to construct it. Each soldier carries with him one article of shelter — his shelter-tent half. To this may be added such other material as can be found.

In wooded countries it is often possible to construct a " lean-to ". These are best made of pine or cedar boughs. The simplest type of a lean-to may be made with the leeward side open. It is a protection against the wind, and a partial protection against snow and rain. The second step in construction consists in building a wall and roof. Walls may be built by piles of stone or by banking sod and earth. The roof may be provided by use of the shelter-tent half, or by crossed support covered with branches and sod. Advantage may be taken of a cliff or a wall. Primitive shelters may be dug into the side of a bank.

Before commencing to build a primitive shelter, first note the direction of the prevailing wind, and build the shelter so that its closed end will be to the windward.

VIII. *Fires.* Men who are inexperienced in wood-craft build their fires too large. An Indian will build a little fire and keep warm, where a white man will build a big fire and remain cold. The body will get more heat from a small fire over which a man can stand, than it will from a large fire from which he must keep away.

It is best to have the fire inside the shelter. The primitive fireplace is built with two walls of sod on either side. It should not be over two feet wide. Its size will depend somewhat upon the kind of fuel which is obtainable. The second step is the construction of a flue, which may be made from stones, sods or green boughs. The side of a bank or cliff against which the shelter is built may form one side of the chimney.

The wood used for fuel must be cut in small pieces. By so doing, its full heating value may be obtained. Fuel is hard to get, must be economized, and must be used to the best advantage.

In every company there are men who have had experience in wood-craft and camp life. Those men must be used as teachers for instruction of those who are ignorant in these matters. They must teach the inexperienced men how to make camp fires, and how to construct for themselves primitive shelters.

IX. *Transportation.* The soldier cannot carry on his back all the weapons he needs to fight the enemy and to fight the cold. During an attack he can take his offensive weapons and enough food, shelter and clothing to enable him to hold his ground for a few hours, until other necessities can be brought up.

The soldier can carry the clothing which he wears, his overcoat, one blanket, and his shelter-tent half. With these a strong and well-nourished man can stand the elements for one day. For a longer time he must have more clothing, better shelter and a fire. This means transportation for blankets, food, fuel, stoves, cooking utensils, and materials for shelter. These materials must be on the ground within twenty-four hours after an advance is made.

Each regiment or unit must have its own supply of cold-weather material. During an attack those articles are to be left under guard at the regimental dump. Wagons and trucks first take food and ammunition to the advancing troops and then return for the cold-weather material, which will enable the men to hold their ground.

*Pneumonias demand as complete isolation* from each other, when of different bacterial etiology, as if they were cases of measles, meningitis or diphteria, all with similar symptoms. Pneumonia, it has been well said, is a group of diseases with certain common signs and symptoms, and never was this more obvious than under present conditions in France, where typical, physical findings and symptoms before death have been found alike in individual patients, and in groups of patients in whom examination of sputum and blood has shown almost pure cultures of pneumococcus of the various strains, streptococcus, influenza bacillus, and meningococcus types B and E, confirmed as well by smears from lungs and blood after death in many instances. It behooves all medical officers to be on their guard against the dangers of mixing or crossing infection among patients by the least neglect of precautions such as cubicle isolation and masking of patients and attendants. In one case recently reported, Type IV pneumococcus and Type E meningococcus were found in abundance in the secretions of different parts of the respiratory tract. The comments of the Commanding Officer of the Army Laboratory are very much to the point, and should stimulate more intensive study of cases and their respective etiology.

" On looking back over the case, there is considerable regret that (*a*) specimens of sputum were not examined at more frequent intervals during the course of the disease; and (*b*) that at the autopsy, smears and cultures were not obtained at varying levels down the entire respiratory tract. "

This case is of chief interest from an epidemiological standpoint, because, if the meningococcus is frequently associated with the pneumococcus (or other organisms), in cases of pneumonia and broncho-pneumonia, aside from its possibilities of being the causative agent of the condition, there arises the important possibility of the wide dissemination of the meningococcus, at the onset of the disease, by coughing and expectoration. This would be especially true in cases of broncho-pneumonia, due to the more gradual onset.

Associated with this case may be mentioned a sharp outbreak of influenza-broncho-pneumonia, occurring in an organization of 329 men; there were 68 cases of this condition, with five deaths within ten days. At the termination of this epidemic of influenza-broncho-pneumonia, a case of epidemic cerebro-spinal-meningitis (Type B, Pasteur), developed in this organization; 29 close contacts with this case of meningitis were cultured (naso-pharynx), with 50 o/o positive carriers, of whom 80 o/o were subsequently shown to be carriers of the meningococcus, Type B, Pasteur. This percentage (50) of positive carriers is considerably in excess of the percentage of positive carriers among contacts with cases of meningitis not associated with coincident respiratory disease, as determined at this laboratory.

*Further evidence* of the distribution of the various pathogenic bacteria in pneumonia cases coming to autopsy is found in the following summary of a report from the Central Laboratory:

"The bacteriological examinations of lungs at autopsies in 30 cases of pneumonia at Base Hospital No. 17, during September, 1918, showed Pfeiffer bacillus in 12 (or 40 o/o), pneumococcus in 13 (or 43 o/o), streptococcus in 10 (or 33 o/o). Considering the difficulties attending the isolation of the influenza bacillus and the possibility of its being absent from the lung at autopsy, although actually an important agent in the disease, the results indicate that this organism has been of primary importance in this group of cases. From other reports of epidemics in various parts of the Advance Section, it is considered that the above findings are characteristic of the present epidemic."

### The Early Diagnosis of Meningitis.

In view of the recent clinical and bacteriological evidence that meningococcemia is the early stage of the infection, later followed by meningeal involvement, the description of the early symptoms upon which a diagnosis may be based, as given by W. W. Herrick, M. R. C. in the *Arch. Int. Med.*, Apr. 15, 1918, is of special interest. If diagnosis can be established while there is yet early meningeal infection, or even in the pre-meningitic stage, intravenous serum therapy will accomplish a correspondingly greater reduction of mortality. Bear in mind that in the A. E. F. only 30.6 o/o of the cases of meningococcus meningitis have returned to duty. The death rate has been 34.2 o/o of the cases. This is a very high mortality rate for adult males of ages 20-40.

The early symptoms are those of a generalized infection. This stage averages about 48 hours, but may be much longer or shorter. The evidences of this stage are (1) Clinical and (2) Bacteriological.

*Clinical Symptoms.* Fever rarely rises above 102 F. (generally ranging from 99 to 101 F.). The pulse is slow with vegal irregularities. There is a characteristic attitude, manner and facies. The patient is not irritable. He resists, prefers to be left alone, lies on his side with thighs and knees drawn up, and shields his face with a covering. He is sensitive to cold and resents exposure. The patient avoids all effort even in speech. He has a vacant look of apathy and dulness.

The veins of the temporal region and forehead are full, the eyeballs are tender, and cyanosis of the face is most common, especially noticeable in the margins of the ears, often with a very sharp line of demarcation. The pupils are almost always dilated. Some cases show retinal edema and obscurations of the disk margins and fulness of the retinal veins.

In many cases there is an upper respiratory tract infection, a coryza, pharyngitis, tonsilitis, pharyngitis; rarely, bronchitis. In four cases a pneumonia was observed. This respiratory involvement is not a mere co-incidence, but demands serious study. Oral secretions are vicid, the tongue coated, and the breath heavy.

Skin manifestations are most important. Very early there may be a more or less diffuse mottling, giving a lead-colored appearance. Tache cerebrale is constant, but of slight diagnostic value. Macular rash, resembling early chicken pox or rose spots, has been observed in a few cases, generally of the less severe type. The predominant skin sign was the petechial rash about the shoulder of the pelvic girdle, and, less frequently, over the trunk extremities.

In connection with the above note on the clinical advantages of early diagnosis of meningitis, special attention is called to the procedure outlined in the " Bulletin on Transmissible Diseases and the Use of Therapeutic Sera " pp. 12-17, prepared by the Director of Laboratories A. E. F. The specifications with regard to the testing of contacts and the determination of carrier rates are particularly valuable.

### Tuberculosis.

Don't take it for granted that every man in the A. E. F. has a right to a little cough. You pick up the man with shrapnel wounds and give him instant care. Tuberculosis is no less disabling. Here is what the past two months have taught us : that the tuberculous soldier is not being found soon enough to give him the best chance of recovery. Most of the cases sent to hospital are moderately advanced or else not tuberculars. Of 176 cases of pulmonary tuberculosis, recently admitted to hospital care, 149 already showed positive sputum. Only 48 had lesions limited to one lung. The average time during which symptoms had been complained of before admission to hospital was 30 days.

Remember that a lung wound of this kind gives no man a right to a chevron on his right coat sleeve.

Pills for complaints of lost weight, chronic cough, a mouthful of blood spat up, loss of " pep " are a kind of conscienceless camouflage. Such patients need a thorough physical examination. The majority of the recent cases of tuberculosis have come from the divisions which were not examined at home by Tuberculosis Boards. The most useful procedure for the detection of apical rales is requiring the patient to breathe in quietly and then out, and, with the last part of the outgoing breath, to give a short, sharp cough. This final expiratory effort and the first of the next inspiration commonly betray the apical lesion, if it exists, by the little shower of minute moist crepitations.

An analysis of the last 63 men with active tuberculosis and positive sputum shows their course by divisions as follows :

| | | | | | |
|---|---|---|---|---|---|
| 26th Division | 10 men | 42nd Division | 6 men | 1st Division | 4 men |
| 41st " | 3 " | 3rd " | 2 " | | |

and one man each from each of the following Divisions : 2, 5, 27, 30, 32, 33, 35, 41, 42, 76, 77, 78, 80, 82, 89, 90, 93, and a few from the labor companies, Stevedore Regiments, etc.

*Pick Them up Promptly*. Through the centuries the bacillus of tuberculosis has established a mode of access to and egress from the human lungs. The desire for a " place in the sun " of this species demands for it new colonies from which to prepare new campaigns. " Tuned up " to the adult lung it is in the soldiers that the achievements of its militarism must be looked up. The bacillus desires to dominate by its " Kultur " and not necessarily to kill. Exhaustion, disease, starvation, and exposure are the allies of this bacillus. Gun shot wounds, the bayonet, and concussions do not assist in the development of tuberculosis; neither to any degree does " gas ". Whereas the general health of the German Army was greatly improved in the second and third years of the war (only one-third as much general sickness as in the first year), tuberculosis greatly increased. 37,000 German soldiers had up to last year been discharged with positive tuberculosis. In war men are " expendable property ", but those with tuberculosis have been badly enough wounded and should be promptly weeded out of the ranks. Read Weekly Bulletin of Disease, Nos. 17, 19, and read also Circular No. 33, C. S. O. for further information. With the exhaustion which many will encounter in the campaigns of this summer, we must look for an increase in the tuberculosis cases. So pick them up promptly.

Most of the Divisions in the American E. F. were weeded of manifest tuberculosis in the U. S. Elimination of tuberculosis from any body of men is like weeding a garden — there is continually a new crop of weeds. The quicker these weeds are plucked, the less the seeds. The shrapnel wounded are picked up quickly, recover, and wear a chevron. The tuberculosis " wounded " are picked up tardily, and receive no honors.

Many divisions contain a medical officer most efficient in the diagnosis of pulmonary tuberculosis. Hunt him out and consult him frequently.

*Le Gérant* : O. Porée. Paris — Imp. Lahure, 9, rue de Fleurus.

